# A Child with an Esophageal Web Treated with Functional Lumen Imaging Probe

**DOI:** 10.1097/PG9.0000000000000304

**Published:** 2023-03-24

**Authors:** Brenna Hohl, Brett Hoskins, Kenneth Ng

**Affiliations:** From the * Department of Medicine, Campbell University School of Osteopathic Medicine, Lillington, NC; † Division of Pediatric Gastroenterology, Hepatology, and Nutrition, Department of Pediatrics, The Johns Hopkins University School of Medicine, Baltimore, MD.

**Keywords:** dilation, EsoFLIP, EndoFLIP, esophageal web, pediatrics, stricture

## Abstract

A 14-month-old male presented to the emergency department with a 4-day history of vomiting after the intake of liquids or solids. During the admission, imaging studies revealed an esophageal web, a form of congenital esophageal stenosis. He was treated with a combination of Endoluminal Functional Lumen Imaging Probe (EndoFLIP) and controlled radial expansion (CRE) balloon dilation, followed by EndoFLIP and EsoFLIP dilation 1 month later. The patient’s vomiting resolved after treatment, and he was able to gain weight. This report describes one of the first cases of applying EndoFLIP and EsoFLIP to treat an esophageal web in a pediatric patient.

## INTRODUCTION

Esophageal stenosis, both congenital and acquired, is rare in the pediatric population, affecting 1 out of every 25 000 live births. Acquired esophageal stenosis can be due to postsurgical healing, esophageal trauma, inflammation, candidiasis, eosinophilic esophagitis, or gastroesophageal reflux disease (GERD) (1). Congenital esophageal stenosis (CES), more commonly seen in the pediatric population, can be described based on pathohistological findings: tracheobronchial remnant (most common), fibromuscular thickening or stenosis, and esophageal web (1). CES often becomes evident as an infant transitions from breastfeeding to semisolid or solid foods, presenting with vomiting and failure to thrive. With severe stenosis, respiratory distress, stridor, cough, and aspiration pneumonia are also possible (2).

Esophageal webs are membranes that remain in the esophagus and have the potential to occlude the lumen of the esophagus, resulting in intermittent dysphagia. Esophageal webs can be seen on fluoroscopic imaging and are confirmed using esophagogastroduodenoscopy (EGD). The etiology of esophageal webs, particularly within the pediatric population, remains largely unknown.

Endoluminal functional lumen imaging probe (EndoFLIP; Medtronic, Minneapolis, MN) is a novel balloon device that uses impedance planimetry to produce real-time quantitative data about the gastrointestinal (GI) lumen, providing important luminal parameters (ie, diameter, cross-sectional area (CSA), compliance, pressure, and distensibility index [DI]) (3). EndoFLIP can be completed in less than 5 minutes during a sedated esophagogastroduodenoscopy (EGD), and can be used to guide endoscopic therapies by providing luminal information before and after treatment. Although EndoFLIP is used for diagnostic evaluation, EsoFLIP (Medtronic) is a therapeutic variant that can also dilate a luminal stricture while also providing the diameter and cross-sectional area in real-time. Although EndoFLIP is currently Food and Drug Administration (FDA) approved in children greater than 5 years of age, Hoskins et al (3) showed that it is also safe when used in children less than 5 years of age. The clinical indication of EndoFLIP is rapidly expanding, especially within the pediatric population.

We describe a 14-month-old male with a congenital esophageal web successfully treated with EndoFLIP and EsoFLIP.

## CASE REPORT

A 14-month-old boy presented to the Johns Hopkins Children’s Center’s emergency department (ED) with a 4-day history of vomiting after eating or drinking. Past medical history was significant for premature birth at 26 weeks gestational age and GERD. A review of systems was notable for decreased activity, choking, vomiting, and decreased urine output. At presentation, he was afebrile and normotensive with age-appropriate heart and respiratory rates, and 100% oxygen saturation. His weight was less than the first percentile on the World Health Organization (WHO) chart. On physical examination, he was tired, fussy, and unable to produce tears while crying. His abdominal examination was unremarkable without distension or tenderness to palpation. Laboratory evaluation was notable for metabolic acidosis with a high anion gap (bicarbonate 15 mmol/L and anion gap 26 mmol/L). Abdominal radiographs showed a nonobstructive bowel gas pattern.

**FIGURE 1.**
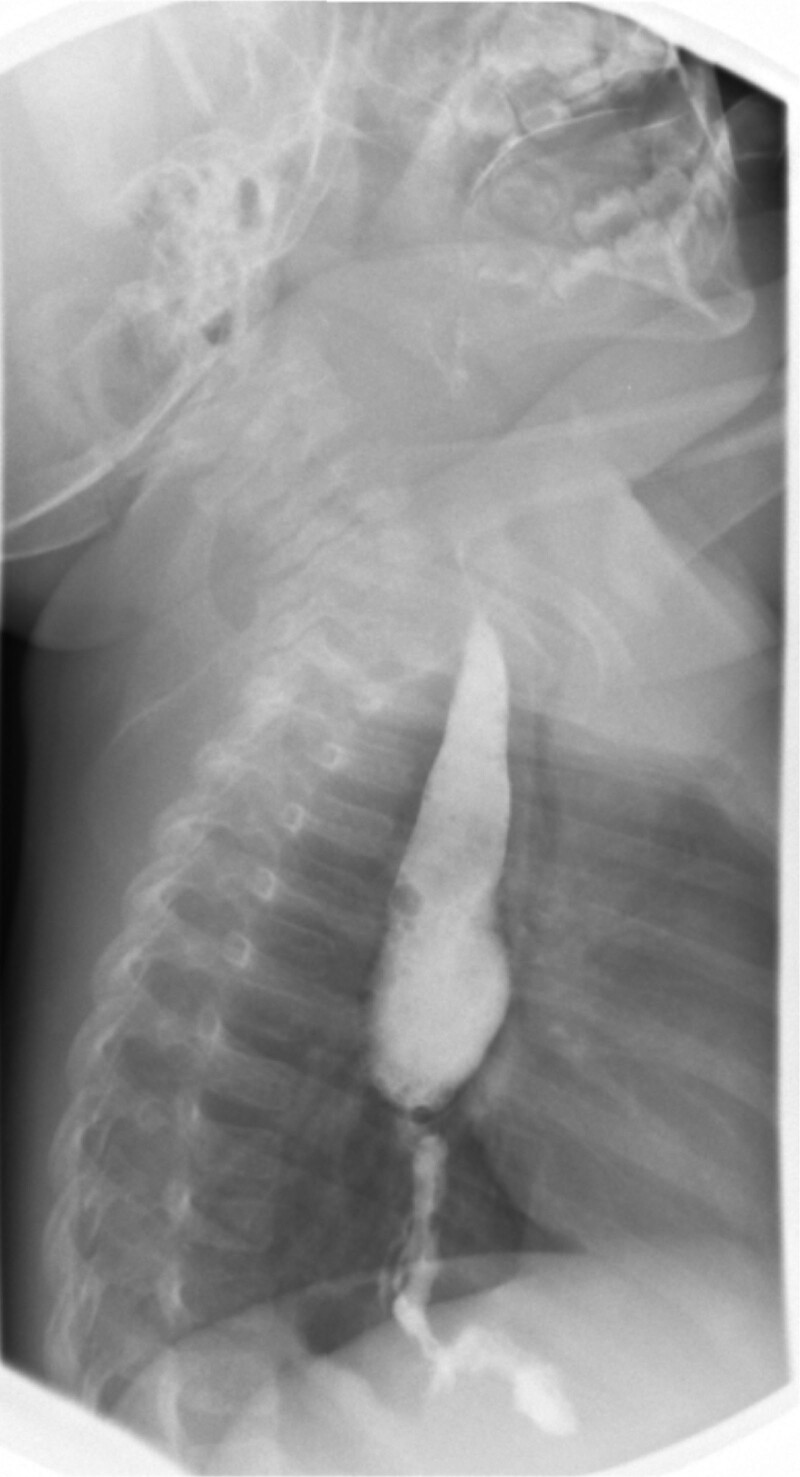
Image from fluoroscopic upper gastrointestinal series showing esophageal stricture.

**FIGURE 2.**
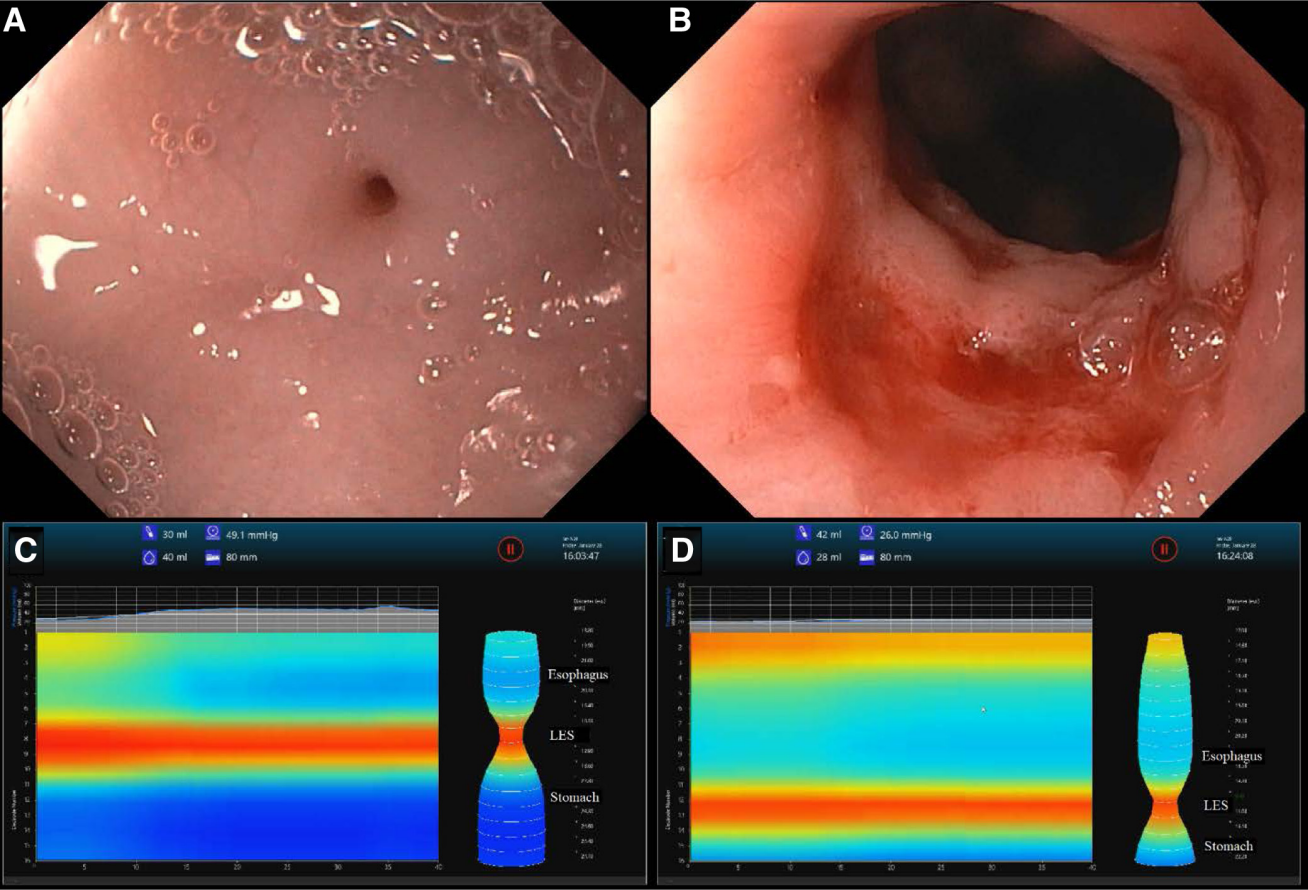
Endoscopic images of the distal esophagus and FLIP topography. A) Significant narrowing of the distal esophagus during the first EGD (pre-dilation). B) Distal esophagus during the third EGD (post-dilation). C) FLIP topography from the third EGD (pre-dilation). D) FLIP topography from the third EGD (post-dilation). EGD = esophagogastroduodenoscopy; FLIP = functional lumen imaging probe.

On admission, a fluoroscopic upper GI series showed an esophageal stricture involving the mid-to-distal esophagus and a small hiatal hernia (Fig. 1). EGD revealed an esophageal web approximately 2 mm in diameter. During the same procedure, guidewire-assisted controlled radial expansion (CRE) balloon dilation was performed using an 8–10 mm balloon. The final diameter of the web was 5.2 mm based on EndoFLIP measurements (Fig. 2 and Table 1). After the procedure, the patient’s vomiting ceased, and he was able to tolerate pureed foods, gaining weight before discharge home.

**TABLE 1.** EndoFLIP measurements taken before and after CRE balloon dilation during the three EGDs

Dilation	Pre- or post- dilation	Inflation (mL)	Diameter (mm)	Compliance (mm^3^/mmHg)	CSA (mm^2^)	DI (mm^2^/mmHg)	Pressure (mmHg)
#1	Predilation	Not collected due to significant esophageal narrowing					
	Postdilation	20	5.1	29.9	20	1.2	16.2
		30	5.2	58.3	21	1.1	19.8
#2	Predilation	20	5.3	81.5	22	1.9	11.3
		30	5.3	52.1	22	0.8	26.2
	Postdilation	20	7.2	97.6	41	3.9	10.6
		30	8.7	92.3	60	2.8	20.9
#3	Predilation	20	6.9	70.3	37	2.3	16.2
		30	8.2	67.1	53	2.0	26.9
	EsoFLIP dilation*	20→30	12.2	-	117	-	-
	Postdilation	20	8.3	115.3	54	4.3	12.6
		30	9.5	98.3	71	3.0	23.9

CRE = controlled radial expansion; CSA = cross-sectional area; DI = distensibility index; EGD = esophagogastroduodenoscopy; EndoFLIP = Endoluminal Functional Lumen Imaging Probe.

*Data obtained during EsoFLIP dilation. The remainder of data in the table was obtained using EndoFLIP.

One month after discharge, a follow-up EGD with EndoFLIP/EsoFLIP was performed, and the web was dilated from 5.3 mm to 8.7 mm. Five months later, he underwent an additional EGD with EndoFLIP/EsoFLIP with successful dilation of the remaining CES from 8.7 mm to 12.2 mm (Fig. 2 and Table 1).

## DISCUSSION

This is one of the first case reports to describe the use of EndoFLIP and EsoFLIP in the management of a pediatric patient with an esophageal web. Our patient had a robust response to treatment and his stricture was successfully dilated from approximately 2 mm to 12.2 mm without complication, which allowed him to safely eat and drink without dietary restriction. Interestingly, the final EndoFLIP measurement showed a diameter of 9.5 mm, which we postulate may be due to postdilation mucosal swelling transiently reducing luminal size. Additionally, the patient’s initial stricture size prevented the use of EndoFLIP and EsoFLIP. This work further supports previously published data that highlights the safety and utility of FLIP devices in the treatment of pediatric esophageal strictures (3). EndoFLIP allows for direct luminal assessment in real time removing the need to rely on fluoroscopic images. EsoFLIP offers a guided, quantifiable dilation without the need for fluoroscopy. We postulate that children with various GI strictures may benefit from EsoFLIP dilation.

This case report also supports the findings from Courbette et al. (4), who described the use of FLIP in the management of pediatric esophageal conditions. The authors showed that FLIP can be used to evaluate and understand various pediatric esophageal disorders in real-time, including before, during, and after dilation (4). Interestingly, the use of FLIP led to a change in the management of 47% of the patients included in their study. Their work showed the utility of FLIP in several GI conditions including achalasia, post-Heller myotomy dysphagia, post-fundoplication dysphagia, and esophagogastric junction outflow obstruction (4).

One limitation of FLIP is that, though it yields objective data, no standardized reference ranges exist for children, leaving interpretation of the results in the hands of clinicians. Additionally, the contraction data generated with the EndoFLIP topography module are based on secondary peristalsis, which is different from the methodology of high-resolution manometry (HRM) (5). However, secondary peristalsis may provide unique advantages, enabling visualization of contractility patterns not seen with HRM (3). Moreover, a recent study by Rosen et al. (6) that compared HRM and FLIP concluded that the two modalities were complementary. Additionally, esophageal strictures that are refractory to endoscopic treatment may require surgical intervention.

EndoFLIP and EsoFLIP should be considered in the management of esophageal webs in children given their ability to provide precise luminal data in real-time, and EsoFLIP’s ability to dilate strictures directly. Future research is needed to establish pediatric-specific reference ranges and to prospectively compare EsoFLIP against traditional dilation modalities.

## ACKNOWLEDGMENTS

Informed consent for case report publication was obtained from the patient’s parents before article submission.
